# Biosynthesis of allene oxides in *Physcomitrella patens*

**DOI:** 10.1186/1471-2229-12-228

**Published:** 2012-11-30

**Authors:** Julia Scholz, Florian Brodhun, Ellen Hornung, Cornelia Herrfurth, Michael Stumpe, Anna K Beike, Bernd Faltin, Wolfgang Frank, Ralf Reski, Ivo Feussner

**Affiliations:** 1 Georg-August-University, Albrecht von Haller Institute for Plant Sciences, Deptartment of Plant Biochemistry, Justus-von-Liebig-Weg 11, 37077, Göttingen, Germany; 2 University of Freiburg, Faculty of Biology, Deptartment of Plant Biotechnology, Schaenzlestrasse 1, 79104, Freiburg, Germany; 3 Ludwig-Maximilians-University Munich, Faculty of Biology, Department Biology I, Plant Molecular Cell Biology, LMU Biocenter, Grosshaderner Str. 2-4, 82152, Planegg-Martinsried, Germany; 4 BIOSS – Centre for Biological Signalling Studies, 79104, Freiburg, Germany; 5 FRIAS – Freiburg Institute for Advanced Studies, 79104, Freiburg, Germany

## Abstract

**Background:**

The moss *Physcomitrella patens* contains C_18_- as well as C_20_-polyunsaturated fatty acids that can be metabolized by different enzymes to form oxylipins such as the cyclopentenone *cis*(+)-12-oxo phytodienoic acid. Mutants defective in the biosynthesis of cyclopentenones showed reduced fertility, aberrant sporophyte morphology and interrupted sporogenesis. The initial step in this biosynthetic route is the conversion of a fatty acid hydroperoxide to an allene oxide. This reaction is catalyzed by allene oxide synthase (AOS) belonging as hydroperoxide lyase (HPL) to the cytochrome P450 family Cyp74. In this study we characterized two AOS from *P. patens*, PpAOS1 and PpAOS2*.*

**Results:**

Our results show that PpAOS1 is highly active with both C_18_ and C_20_-hydroperoxy-fatty acid substrates, whereas PpAOS2 is fully active only with C_20_-substrates, exhibiting trace activity (~1000-fold lower k_cat_/K_M_) with C_18_ substrates. Analysis of products of PpAOS1 and PpHPL further demonstrated that both enzymes have an inherent side activity mirroring the close inter-connection of AOS and HPL catalysis. By employing site directed mutagenesis we provide evidence that single amino acid residues in the active site are also determining the catalytic activity of a 9-/13-AOS – a finding that previously has only been reported for substrate specific 13-AOS. However, PpHPL cannot be converted into an AOS by exchanging the same determinant. Localization studies using YFP-labeled AOS showed that PpAOS2 is localized in the plastid while PpAOS1 may be found in the cytosol. Analysis of the wound-induced *cis*(+)-12-oxo phytodienoic acid accumulation in *PpAOS1* and *PpAOS2* single knock-out mutants showed that disruption of PpAOS1, in contrast to PpAOS2, results in a significantly decreased *cis*(+)-12-oxo phytodienoic acid formation. However, the knock-out mutants of neither *PpAOS1* nor *PpAOS2* showed reduced fertility, aberrant sporophyte morphology or interrupted sporogenesis.

**Conclusions:**

Our study highlights five findings regarding the oxylipin metabolism in *P. patens*: (i) Both AOS isoforms are capable of metabolizing C_18_- and C_20_-derived substrates with different specificities suggesting that both enzymes might have different functions. (ii) Site directed mutagenesis demonstrated that the catalytic trajectories of 9-/13-PpAOS1 and PpHPL are closely inter-connected and PpAOS1 can be inter-converted by a single amino acid exchange into a HPL. (iii) In contrast to PpAOS1, PpAOS2 is localized in the plastid where oxylipin metabolism takes place. (iv) PpAOS1 is essential for wound-induced accumulation of *cis*(+)-12-oxo phytodienoic acid while PpAOS2 appears not to be involved in the process. (v) Knock-out mutants of neither AOS showed a deviating morphological phenotype suggesting that there are overlapping functions with other Cyp74 enzymes.

## Background

Oxidized fatty acids that are collectively termed oxylipins exhibit signaling functions in fungi [1,2], mammals [3] and flowering plants [4]. Also non-flowering plants like mosses have been shown to contain oxylipins, but knowledge on their physiological role is still scarce [5]. In flowering plants the oxylipin derivative jasmonic acid (JA) regulates developmental processes as well as defense responses [6,7]. This plant hormone is synthesized via the so-called allene oxide synthase (AOS) branch of the oxylipin pathway that takes place in two spatially separated cell compartments [8]: It may start in the plastid with the release of 18:3(n-3) (or in some plants also with 16:3(n-3)) from a membrane lipid by the action of a lipase [9]. The free fatty acid may then be oxidized by a specific 13*S*-lipoxygenase (13*S*-LOX) yielding 13*S*-hydroperoxy octadecatrienoic acid (13*S*-HPOT(n-3)). This product serves as substrate for further sequential reactions that are catalyzed by two enzymes: the AOS transforms 13*S*-HPOT(n-3) in a first reaction step to a highly unstable allene oxide which hydrolyses in aqueous solution within 30 seconds yielding α-ketol and γ-ketol as well as cyclopentenone derivatives [10,11]. In the presence of the second enzyme, the allene oxide cyclase (AOC), however, the allene oxide is cyclized to enantiopure *cis*(+)-12-oxo phytodienoic acid (*cis*(+)-OPDA) [12]. *cis*(+)-OPDA is transported into the peroxisome where it is reduced to the respective cyclopentanone derivative [13] and further processed by three rounds of β-oxidation finally yielding (+)-7-iso-JA [14,15].

**Figure 1:**
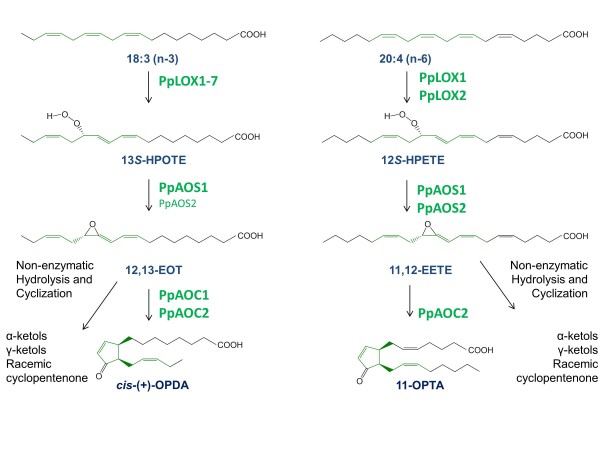
**Overview of the oxylipin biosynthesis pathways in *P. patens *(modified from [**[22]**]).** 18:3(n-3) may be oxidized by one of the seven identified LOXes yielding 13-HPOTE(n-3). This compound is specifically dehydrated by PpAOS1 to 12,13-epoxy octadecatrienoic acid. The allene oxide is unstable and hydrolyses in aqueous solution non-enzymatically to α- and γ-ketols or cyclizes to a racemic mixture of 12-oxo phytodienoic acid (OPDA). In the presence of PpAOC1/2, however, enantiopure *cis*(+)-OPDA is formed. Analogous reactions are starting from 20:4(n-6) that is converted by PpLOX1/2 to 12-HPETE and further dehydarated by PpAOS1/2 yielding the unstable allene oxide derivative 11,12-epoxy eicosatetraenoic acid (11,12-EETE), which can be also non-enzymatically converted to the respective α- and γ-ketol derivatives or racemic cyclopentenones. Only in the presence of PpAOC2 formation of enantiopure 11-oxo prostatrienoic acid (11-OPTA) is possible.

Over the last years several studies in the moss *Physcomitrella patens* described an oxylipin pathway analogous to that of flowering plants [16-19]. However, in contrast to flowering plants this moss contains besides C_18_- also C_20_-polyunsaturated fatty acids which are typically found in mammalian cells. Further, *P. patens* is able to metabolize both groups yielding a set of diverse oxylipins (Figure 1). In an initial reaction step two unusual bifunctional LOXs may oxidize 20:4(n-6) at the C-12 yielding 12-hydroperoxy eicosatetraenoic acid (12-HPETE) [17,20]. This product serves as a substrate for at least three enzyme reactions that may lead to the formation of different C_8_- and C_9_-products: these volatiles are either formed by the hydroperoxide cleaving activity of the bifunctional LOXs [17,19] or by a classical hydroperoxide lyase (HPL) belonging to the Cyp74 enzyme family [18,19]. On the other hand 12-HPETE can also be dehydrated by PpAOS yielding 11,12-epoxy eicosatetraenoic acid (Figure 1) [16,21]. In analogy to the clas-sical octadecanoid pathway, this unstable allene oxide is re-arranged by one particular AOC (PpAOC2) forming 11-oxo prostatrienoic acid (11-OPTA) [16]. The molecular basis for this distinct substrate specificity of PpAOC2 and the mechanism of the cyclization reaction catalyzed by PpAOC1 and 2 has recently been investigated by X-ray crystallography [22]. Interestingly, recent studies demonstrated that upon wounding and pathogen attack only *cis*(+)-OPDA, but no JA accumulated in *P. patens*. These findings led to the hypothesis that, in contrast to higher plants, *P. patens* harbors only the plastid-localized part of the oxylipin pathway, while the peroxisomal part is missing [16,23]. In line with this assumption were immunocytological investigations that demonstrated the plastidic localization of PpLOX and PpAOC [16].

AOS and HPL together with two further members, divinylether synthase (DES) and epoxyalcohol synthase, belong to the cytochrome P450 subfamily Cyp74 [24-26]. In contrast to classical P450s these enzymes do not act as monooxygenases but rather as hydroperoxide isomerases (HPL) or dehydratases (AOS and DES) [27]. Consequently, since they use fatty acid hydroperoxides as their natural substrate, Cyp74s - in contrast to classical P450s - do neither need molecular oxygen nor external electron donors for catalysis. The 3D-structures of two AOSs from Arabidopsis and Guayule demonstrated that these functional differences in Cyp74- and P450-catalysis can be explained by different active site architectures that preclude monooxygenase-activity from those unusual P450s [25,28]. Despite this progress in understanding the underlying structure-function relationship, there are mechanistic aspects that are still not fully understood. Recently, it has been established that the catalytic trajectories of different Cyp74s are closely related [29] and single point mutations may be sufficient to inter-convert Cyp74-activities [25,27,30,31]. For example, for AtAOS it was demonstrated that mutation of one particular phenylalanine residue (Phe-137) which may be responsible for stabilizing intermediately formed carbon centered substrate radicals converts AOS into HPL activity [25,30]. In addition, the close interconnection of both trajectories is also mirrored by mutational studies that identified two conserved amino acids in the I-helix region of AOS. Substitution of this Phe and Ser (Phe-295 and Ser-297 in tomato AOS3) by Ile and Ala, respectively, led to an enzyme with HPL-activity [31]. Similar results were obtained when Lys-302 and Thr366 were exchanged in tomato AOS3 by Ser and Tyr, respectively [27].

In the present study we identified an additional AOS enzyme, PpAOS2, and analyzed the biochemical properties of Cyp74-enzymes from *P. patens*. We established a recombinant *E. coli* expression system that enabled us to produce and purify PpAOS1, PpAOS2 as well as PpHPL in high amounts and to analyze a set of different biochemical parameters and compare those for the different enzymes. By employing site directed mutagenesis we provide further evidence that the inter-conversion of Cyp74-activites by specific single amino acid exchanges can also be applied on substrate unspecific AOS. Besides the molecular details of Cyp74-catalysis, we also aimed to analyze the sub-cellular localization and physiological function of PpAOS1 and PpAOS2. Localization studies using YFP-labeled AOS demonstrated that PpAOS2 is localized in the plastid while PpAOS1 is only detected within the cytosol. Interestingly, the knock-out mutants of neither PpAOS1 nor PpAOS2 showed a morphological phenotype deviating from wild type.

## Results

### Identification of a third Cyp74 enzyme from *P. patens*

**Figure 2:**
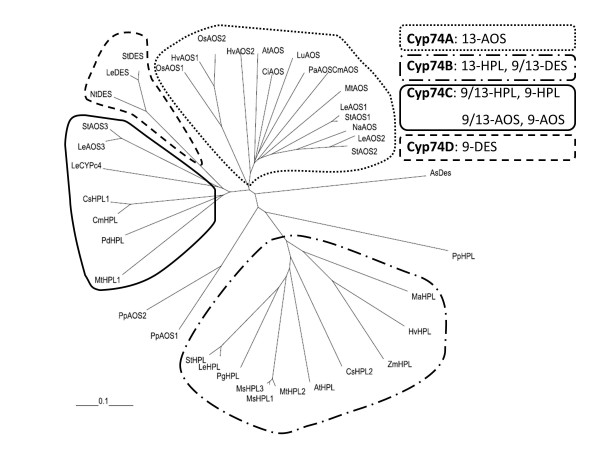
**Phylogenetic analysis of different Cyp74 enzymes from different plant species: *Allium sativum *(As), *Arabidopsis thaliana *(At), *Citrus sinensis *(Ci), *Cucumis melo *(Cm), *Cucumis sativum *(Cs), *Hordeum vulgare *(Hv), *Lycopersicum esculentum *(Le), *Musa ascuminata *(Ma), *Medicago sativum *(Ms); *Medicago truncatula *(Mt); *Nicotiana attentuata *(Na), *Nicotiana tabacum *(Nt), *Oryzae sativum *(Os), *Parthenium argentatum *(Pa), *Prunus dulcis *(Pd), *Psidium guajava *(Pg), *Physcomitrella patens *(Pp), *Solanum tuberosum *(St), *Zea mays *(Zm), AtAOS, CAA63266; AsDES, AJ867809; AtHPL, AAC69871; CiAOS, AA072741; CmAOS, AAM66138; CmHPL, AAK54282; CsHPL1, AAF64041; CsHPL2, AF229812; HvAOS1, CAB86384; HvAOS2, CAB86383; HvHPL, CAC82980; LeAOS1, CAB88032; LeAOS2, AAF67141; LeAOS3, AAN76867; LeCYPc4, AAL86702; LeDES, AAG42261; LeHPL, AAF67142; LuAOS, AAA03353; MaHPL, CAB39331; MsHPL1, CAB54847; MsHPL2, CAB54848; MsHPL3, CAB54849; MtAOS, CAC86897; MtHPL2, CAC86899; MtHPL1, CAC86898; NaAOS, CAC82911; NtDES, AAL40900; OsAOS1, AY055775; OsAOS2, AAL38184; PaAOS, CAA55025; PdHPL, CAE18065; PgHPL, AAK15070; PpAOS2, XP_001759629; PpAOS1,CAC86919; PpHPL, CAC86920; StAOS1,CAD29735; StAOS2, CAD29736; StAOS3, CAI30876; StDES, CAC28152; StHPL, CAC44040; ZmHPL, AAS47027.** The phylogenetic tree was calculated using the ClustalX software package employing default parameters.

Previously two Cyp74 enzymes were identified from *P. patens*, a HPL (PpHPL) [18] and an AOS (PpAOS1) [21]. Further analysis of EST sequences and the genome of *P. patens* revealed the existence of a third putative Cyp74 enzyme. By sequence homology it was supposed to be also an AOS, named PpAOS2. Sequence alignments of Cyp74s from different plants with the *P. patens* enzymes showed that similar to PpHPL [18] also both AOS isoforms (PpAOS1 and PpAOS2) contain sequence motifs characteristic for members of the Cyp74-family [25]. Besides the ExxR motif that is typical for all P450-enzymes [32], the three sequences also include the distinctive nine amino acid insert in the heme signature motif harboring the essential cysteine residue that serves as the 5^th^ heme ligand [25]. As has been observed for PpHPL, a phylogenetic analysis shows that all Cyp74 from *P. patens* do not group with other members of different Cyp74-subfamilies from flowering plants (Figure 2) [18], suggesting that there are significant differences in their amino acid sequences. In order to verify the tentative identification of PpAOS2 as AOS, we cloned both PpAOS isoforms and expressed them in addition to PpHPL in *E. coli*.

### Cloning and expression

**Figure 3:**
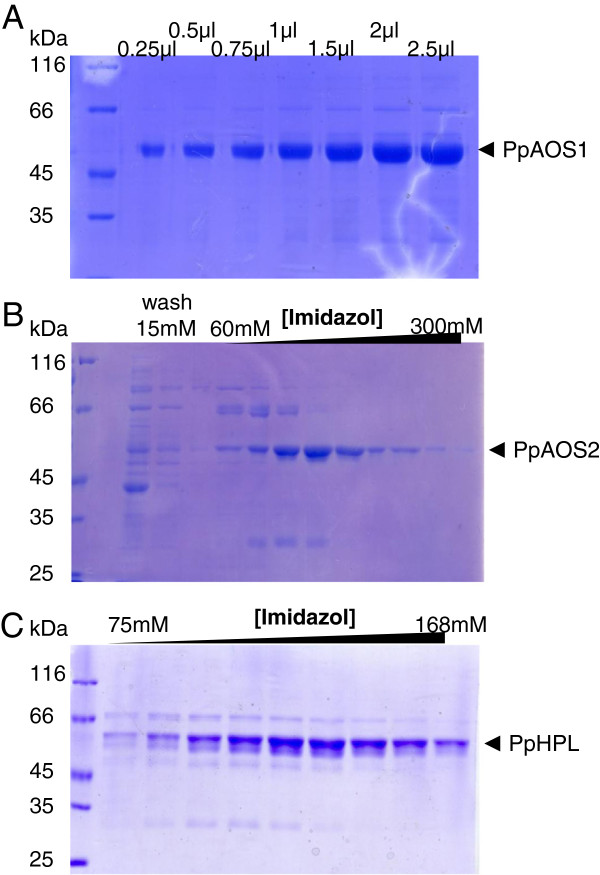
**SDS-PAGE analysis of purified PpAOS1 (A), PpAOS2 (B) and PpHPL (C).** All enzymes were expressed as His-tagged proteins and purified via Ni^2+^-affinity chromatography. Note that different fractions of final elution employing a linear gradient with increasing imidazol concentration are shown in (**B**) and (**C**). In case of PpAOS2-purification we applied an additional washing step, in which we washed the column with 50 mM sodium phosphate buffer (pH 8.0) containing 50 mM NaCl, 500 mM urea and 15 mM imidazol in order to further elute unspecifically bound proteins as shown in (**B**).

Both AOS genes were PCR-amplified from a cDNA library of *P. patens* protonema and were expressed in *E. coli* in frame with a N-terminal hexahistidine peptide. In order to improve the protein yield of PpHPL, we added the MAKKTSS-sequence that has been used previously for improving the solubility of AtAOS [25]. The resulting fragment was re-cloned in frame with a C-terminal hexahistidine sequence and expressed in *E. coli*. Protein extraction was essentially performed as reported before [33]. By this PpAOS1 and PpAOS2 as well as PpHPL could be purified nearly to homogeneity (Figure 3).

**Figure 4:**
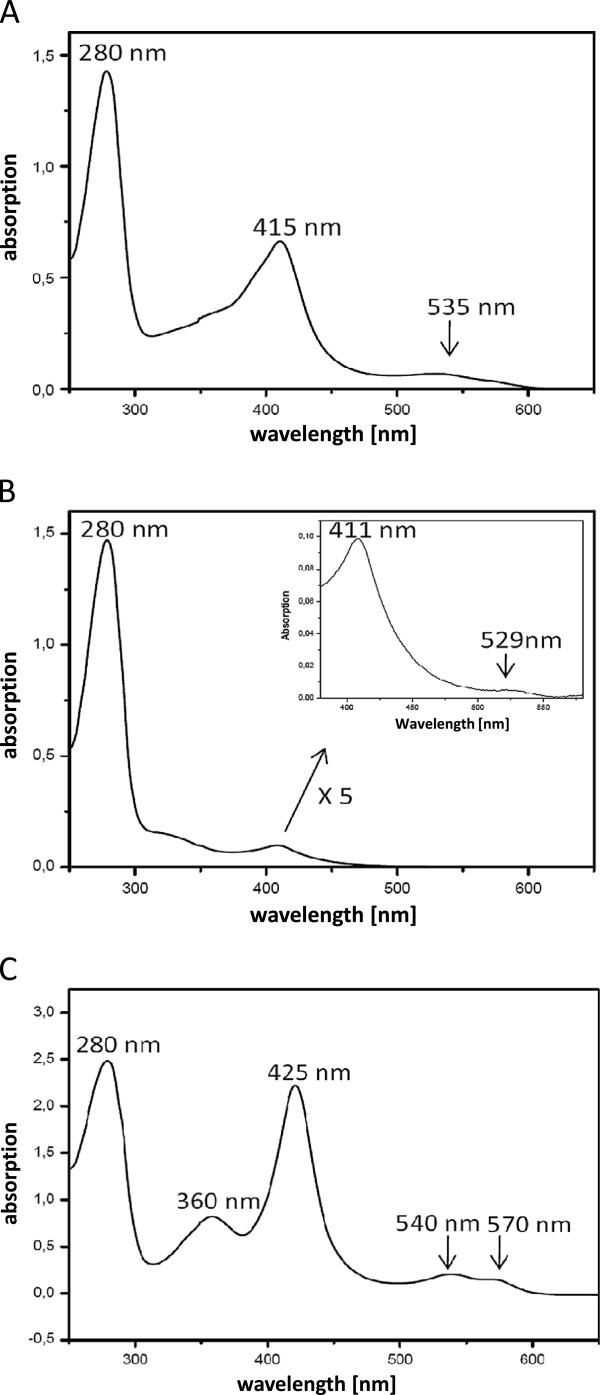
**UV/vis spectra of purified PpAOS1 (A), PpAOS2 (B) and PpHPL (C).** All spectra were measured in 100 mM sodium phosphate buffer (pH 6.0).

Throughout all preparations, buffers showed a reddish color which suggested the presence of a heme co-factor. Indeed, when UV/vis spectra of PpAOS1, PpAOS2 and PpHPL were recorded, in every case an absorption profile that is characteristic for heme proteins was detectable: While the UV/vis spectra showed distinctive heme absorption maxima at 415 nm (Soret-band), 535 nm (β-band) for PpAOS1 and 411nm (Soret-band) and 529 nm (β-band) for PpAOS2 (Figure 4A and B), PpHPL had absorption maxima at 425 nm (γ, Soret), 360 nm (δ), 540 nm (β) and 570 nm (α) (Figure 4C). It should be noted at this point, that the heme occupancy of both PpAOS-isoforms was significantly reduced: Based on theoretical molar extinction coefficients at 280 nm (ε_280_(PpAOS1) ≈ 62000 M^-1^ cm^-1^ and ε_280_(PpAOS2) ≈ 57000 M^-1^ cm^-1^; as calculated with the Protparam-software tool (http://web.expasy.org/protparam/)) and an expected molar extinction coefficient for the Soret-band of ε_Soret_ ≈ 100 000 M^-1^ cm^-1^, we calculated a heme-content of PpAOS1 of approx. 30% while that of PpAOS2 was only 4%.

### pH-optimum and kinetic properties

In order to identify the optimal pH of PpAOS1 and PpAOS2, the conversion of 13*S*-hydroperoxy octadecadienoic acid (13-HPOD) was followed photometrically at 234 nm in dependence on different pH values. While PpAOS1 showed the highest activity around pH 6.5, the optimal pH for PpAOS2 was at pH 7.0.

Kinetic properties were analyzed in analogous experiments in which the catalytic activity was measured in dependence on the substrate concentration at the respective optimal pH value. As substrates we used the 9 and 13-hydroperoxides derived from 18:2(n-6), 9-/13-HPOD, 18:3(n-6), 9/13-HPOT(n-6), 18:3(n-3), 9/13-HPOT(n-3), as well as the 12-hydroperoxy derivative of 20:4(n-6), 12-HPETE. Kinetic constants that were determined by plotting the experimentally derived data points to the Michaelis-Menten equation are summarized in Table 1. Exemplarily shown in Additional file 1: Figure S1 are the Michaelis-Menten plots for the conversion of 9-HPOT(n-3) by PpAOS1 (Additional file 1: Figure S1A) and PpAOS2 (Additional file 1: Figure S1B). The given k_cat_-values were calculated by taking into account that the heme occupancy of both PpAOS were 30% and 4%, respectively (see above). Based on the k_cat_/K_M_-values, these results show that PpAOS1 isomerizes 9- and 13-hydroperoxides derived from 18:2(n-6), 18:3(n-6) and 18:3(n-3) with a similar specificity as 12-HPETE. Interestingly, the kinetic data showed that in contrast to PpAOS1, PpAOS2 (Table 2) has a distinct specificity for 12-HPETE; while this substrate was specifically converted with a k_cat_/K_M_ of 1229 × 10^6^ min^-1^ M^-1^ the C_18_-hydroperoxy derivatives were only very poor substrates with k_cat_/K_M_-values below 1 × 10^6^ min^-1^ M^-1^.

**Table 1:** Kinetic properties of PpAOS1 with different hydroperoxy fatty acid substrates

**Substrate**	**K**_**M**_**[μM]**	**V**_**max**_**[μM/min]**	**k**_**cat**_**[1/min]**	**k**_**cat**_**/K**_**M**_**[min**^**-1**^**M**^**-1**^**× 10**^**6**^**]**
9-HPOD	121 +/− 61	0.98 +/− 0.29	32680	270
9- HPOT(n-3)	39 +/− 14	0.20 +/− 0.03	6706	172
9-HPOT(n-6)	46 +/− 17	2.35 +/− 0.37	156733	3426
13-HPOD	83 +/− 42	0.96 +/− 0.27	31856	384
13-HPOT(n-3)	95 +/− 27	0.90 +/− 0.16	30070	316
13-HPOT(n-6)	107 +/− 57	2.77 +/− 0.88	184500	1731
12-HPETE	7 +/− 2	0.23 +/− 0.02	7657	1176

Kinetic properties were determined by measuring the initial time-dependent substrate consumption at 234 nm at different substrate concentrations typically ranging from 2–100 μM. In some cases the highest substrate concentration applied was 150 μM. For analysis between 20 and 30 data points were fitted to the Michaelis-Menten equation. Note that PpAOS1-concentrations used for incubations with 9- and 13-HPOT(n-6) were different (0.05 nM) from those used for incubations with the other substrates (0.1 nM). K_cat_ values are corrected to 100% heme occupancy from the ~30% heme content in the enzyme preparation.

**Table 2:** Kinetic properties of PpAOS2 with different hydroperoxy fatty acid substrates

**Substrate**	**K**_**M**_**[μM]**	**V**_**max**_**[μM/min]**	**k**_**cat**_**[1/min]**	**k**_**cat**_**/K**_**M**_**[min**^**-1**^**M**^**-1**^**× 10**^**6**^**]**
9-HPOD	36 +/− 5	0.02 +/− 0.001	5	0.14
9- HPOT(n-3)	40 +/− 4	0.01 +/− 0.001	2.5	0.06
9- HPOT(n-6)	28 +/− 4.	0.03 +/− 0.002	7.5	0.27
13-HPOD	27+/− 3	0.04 +/− 0.002	10	0.37
13- HPOT(n-3)	30 +/− 5	0.02 +/− 0.002	5	0.17
13- HPOT(n-6)	42 +/− 10	0.02 +/− 0.002	5	0.12
12-HPETE	10 +/− 5	0.49 +/− 0.057	12250	1228.69

Kinetic properties were determined by measuring the initial time-dependent substrate consumption at 234 nm at different substrate concentrations typically ranging from 2–100 μM. For analysis between 20 and 30 data points data points were fitted to the Michaelis-Menten equation. Note that the PpAOS2-concentration used for incubations with 12-HPETE was different (1 nM) from those used for incubations with the other substrates (100 nM). K_cat_ values are corrected to 100% heme occupancy from the ~4% heme content in the enzyme preparation.

### Product analysis and site directed mutagenesis

**Figure 5:**
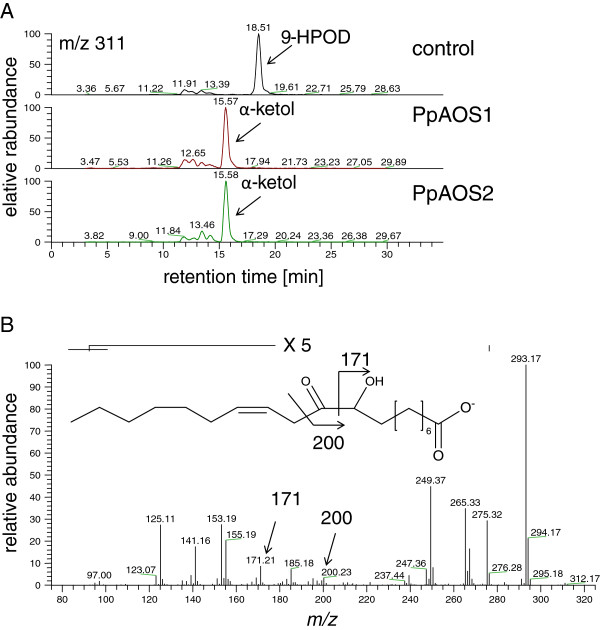
**LC/MS-analysis of products formed from 9-HPOD by incubation with PpAOS1 and PpAOS2, respectively.** (**A**) Shown is an extracted ion chromatogram (*m/z* 311) of the RP-HPLC/MS-analysis of products derived from incubation of 9-HPOD with reaction buffer (control), PpAOS1 and PpAOS2. (**B**) The tandem-MS spectrum of the major peak shown in (A), which is in accordance to those reported earlier for α-ketol derivatives [34,35].

As activity of PpAOS2 with C_18_-hydroperoxy fatty acids is very low, we decided to verify the enzymatic properties of the AOS towards these substrates by analyzing the produced ketol derivatives via LC-MS. As substrate we chose 9*S*-hydroperoxy octadecadienoic acid (9-HPOD) and compared product formation after incubation with PpAOS2 as well as PpAOS1. As shown in Figure 5 these analyses demonstrated that indeed both enzymes (PpAOS1 and 2) are AOS, since they both formed α-ketols from 9-HPOD.

**Figure 6:**
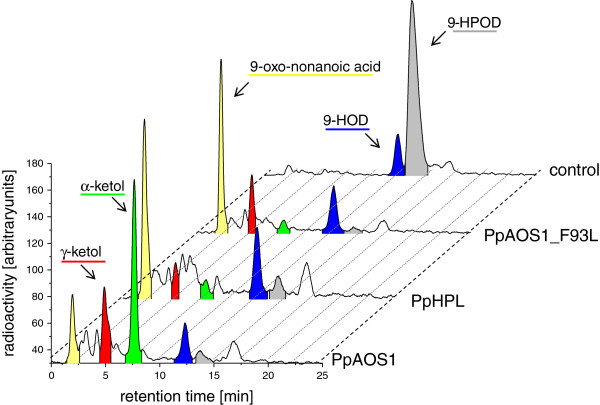
**Analysis of products formed from incubation of [1-**^**14**^**C]-9-HPOD with PpHPL, PpAOS1 and PpAOS1_F93L, respectively.** Purified enzymes were incubated with radio-labeled substrate and incubated for 30 min. After extractive isolation, products were analyzed by means of RP-HPLC coupled to a radio-detector.

Subsequently, molecular determinants that may be essential for the activity of HPL and AOS from *P. patens* were analyzed. The focus was on PpAOS1 and PpHPL, because those enzymes showed, in contrast to the well studied AOS from *A. thaliana*[36,37], a broad substrate specificity metabolizing 9 as well as 13-hydroperoxy fatty acid derivatives. Using these enzymes we wanted to address the question whether the determinants reported for 13-hydroperoxide specific AtAOS also influence the catalysis of unspecific AOS enzymes. For this, we generated a set of different PpAOS1 and PpHPL variants and analyzed the product specificity with the 9- and 13-hydroperoxy isomers of 18:2(n-6) and 18:3(n-3) by RP-HPLC. A representative example is shown in Figure 6. Incubations of 9-HPOD with PpAOS1_WT yielded besides α- and γ-ketols, which are derived from a non-enzymatical hydrolysis of the allene oxide product, also significant amounts of the characteristic HPL product, 9-oxo nonanoic acid. On the other hand, analogous incubations demonstrated that purified PpHPL catalyzed the conversion of 9-HPOD to the main product 9-oxo nonanoic acid as well as to a set of different site products *inter alia* α- and γ-ketols. Similar results were obtained when 9-, 13-HPOT or 13-HPOD were used as substrate (Table 3). These results are consistent with a close interconnection of both enzymatic pathways as proposed before [25,29,30].

**Table 3:** Product specificities of different PpAOS1 and PpHPL variants

**Substrate**	**Enzyme-variant**	**ω-oxo fatty-acids (= HPL activity)**	**Ketols (= AOS activity)**	**Cyclopentenone (= AO cyclization)**
9-HPOD	HPL Wt	++++	+	n.d.
	AOS1 Wt	+	++++	n.d.
	AOS1 F93L	++++	+	n.d.
9-HPOT(n-3)	HPL Wt	++++	+	n.d.
	HPL F151L	++++	+	n.d.
	HPL A169S	++++	+	n.d.
	HPL F151L, A169S	++++	+	n.d.
	AOS1 Wt	+	++++	n.d.
	AOS1 F93L	++++	-	n.d.
13-HPOD	HPL Wt	++++	+	n.d.
	HPL F151L	++++	+	n.d.
	HPL A169S	++++	+	n.d.
	HPL F151L, A169S	++++	+	n.d.
	AOS1 Wt	+	++++	n.d.
	AOS1 F93L	++++	+	n.d.
13-HPOT(n-3)	HPL Wt	++++	+	n.d.
	HPL F151L	++++	+	n.d.
	HPL A169S	++++	+	n.d.
	HPL F151L, A169S	++++	+	n.d.
	AOS1 Wt	+	+++	+
	AOS1 F93L	++	++	+

Affinity purified enzymes were incubated with [1-^14^C]-labeled hydroperoxy fatty acids for approx. 30 min. After extraction products were analyzed by RP-HPLC that was coupled to a radio-detector and quantified by integration of the respective peak area. For simplicity the relative amounts of each product is indicated by the number of “+”. AOS, allene oxide synthase; HPL, hydroperoxide lyase; Wt, wild type; n.d., not determined. The data represent between 2 and 5 independent experiments.

Sequence alignments showed that both AOS isoforms from *P. patens* contain the strictly conserved Phe residue (Phe-93 in PpAOS1 and PpAOS2) in the active site. This residue is essential for AOS activity by stabilizing the intermediately formed carbon-centered substrate radicals. In case of HPL this particular position is normally occupied by a Leu residue. In contrast to Phe this residue is unable to stabilize reactive product intermediates and thus leads to the formation of an unstable hemiacetal which decomposes spontaneously to short chain aldehydes [38]. Consequently, incubation of 9-HPOD with the respective PpAOS1 variant, in which Phe-93 was substituted by Leu, resulted in an increased formation of 9-oxo nonanoic acid and a concomitant decrease in the amount of α-ketols (Figure 6). Incubations with 13-HPOD as well as with the respective hydroperoxy isomers of 18:3(n-3), respectively, gave similar results (Table 3), suggesting that this residue is an essential determinant for both 9- and 13-hydroperoxide dehydration. Interestingly, sequence alignments demonstrated that PpHPL also has a Phe at this particular position. In order to analyze the significance of this residue for HPL activity, we substituted Phe- 151 by Leu. As shown in Table 3 for all substrates tested, this variant showed an unaltered activity and formed a similar set of products as the wild type enzyme. A similar result was obtained when we substituted a conserved HPL-specific Ala (Ala-169) in the active site by an AOS-specific Ser – a residue that additionally has been reported to determine AOS activity. Neither the single variant (PpHPL_A169S) nor the double variant (PpHPL_F151L/A169S) showed an altered catalytic specificity (Table 3).

### Intercellular localization of PpAOS1 and PpAOS2

**Figure 7:**
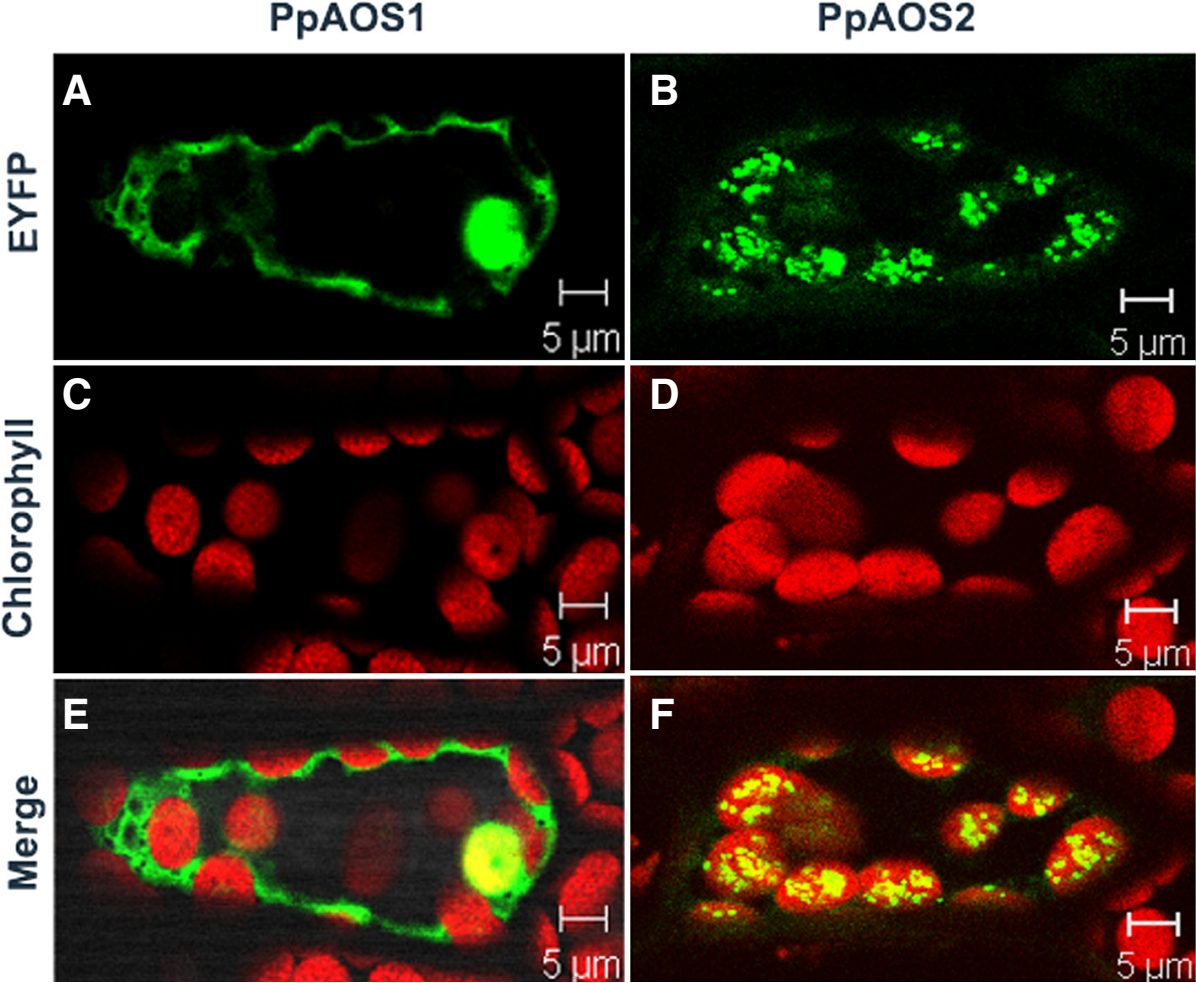
**Subcellular localization of PpAOS1 and PpAOS2.***P. patens* gametophytes were transfected with constructs for expressing C-terminal YFP-labeled PpAOS1 and PpAOS2. YFP-fluorescence of PpAOS1-YFP and PpAOS2-YFP is shown in **A** and **B**, respectively. Chlorophyll auto-fluorescence is shown in **C** and **D** whereas a merged image of A and C as well as B and D is shown is in **E** and **F**, respectively.

Recently, PpHPL was shown to be associated with plastidic membranes by transient expression of a PpHPL-YFP fusion protein in *P. patens* protoplasts [18]. In line with this are bioinformatic analyses that predicted a plastidic transit peptide sequence at the N-terminus of PpHPL. In neither PpAOS1 nor PpAOS2 a plastidic transit peptide sequence was identified using pSORT (http://psort.nibb.ac.jp/form.html), TargetP (http://www.cbs.dtu.dk/services/TargetP/) or WoLF PSORT (http://wolfpsort.org/). In order to analyze the intracellular localization of both AOS isoforms, C- terminal YFP-fusions were generated and transiently expressed in *P. patens* gametophores. In case of PpAOS1, YFP fluorescence was distributed within the cytosol, whereas PpAOS2-YFP co-localized with red chlorophyll auto fluorescence of chloroplasts (Figure 7). To confirm the localization results we also generated vectors for expression of PpAOS1 and PpAOS2 with a C-terminal CFP-fusion and obtained similar results (data not shown). In addition, we performed transient expression studies with all YFP and CFP fusion constructs in onion epidermis cells. We detected cytosolic localization for PpAOS1-fusions, while for PpAOS2-fusions fluorescence was observed only in granular structures of plastids (Figure 7). Additional attempts to express PpAOS1 and PpAOS2 in *P. patens* protoplasts gave the same results (data not shown). However, the analysis of the import of *in vitro*-translated PpAOS1 and 2 protein into isolated pea protoplasts failed. Thus, in contrast to most other Cyp74 enzymes our results suggest that PpAOS1 may be localized in the cytosol and only PpAOS2 is localized within the plastid. It should be noted in this context, that similar to what was reported for PpHPL [18], PpAOS2 may not be equally distributed in the chloroplast as a rather in-homogenous clustering of PpAOS2 was observed.

### Analysis of *P. patens* Δ*PpAOS1* and Δ*PpAOS2*- knock-out mutants

**Figure 8:**
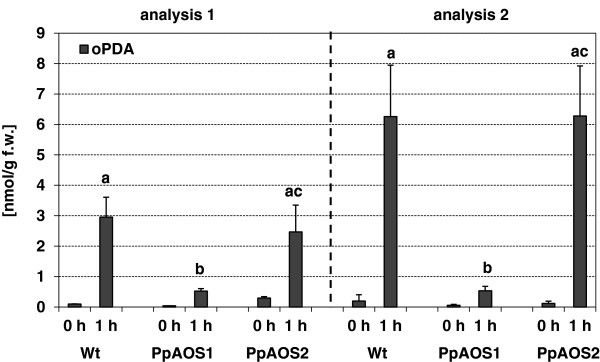
**Analysis of *cis*(+)-OPDA in *P. patens *WT, *ΔPpAOS1 *and *ΔPpAOS2.****cis*(+)-OPDA was extracted from unwounded (control) and wounded (1h after wounding stimulus) moss by employing the methyl-tert-butyl ether technique [41] and analyzed via LC/MS [42]. Shown are the results from two independent experimental datasets. Data are presented as mean values with standard deviations from two - six biological replicates. Values with significant differences (Students *T*-Test; P < 0.05) are indicated above the respective column by different letters.

Subsequently, the physiological role of PpAOS1 and PpAOS2 was analyzed. Former studies on **Δ***PpAOC* knock-out mutants showed reduced fertility, aberrant sporophyte morphology and interrupted sporogenesis [16]. To investigate if the deletion of *PpAOS1* and *PpAOS2* cause similar effects on reproduction in *P. patens* we generated targeted knock-out mutants of *PpAOS1* and *PpAOS2*[39]. Two independent mutant lines with a disrupted *PpAOS1* locus (Δ*PpAOS1***)** and five independent mutant lines with a disrupted *PpAOS2* locus (Δ*PpAOS2***)** were identified by PCR. Gametophores of all knockout lines were grown for four weeks under standard conditions which led to the formation of colonies. We did not observe any differences during vegetative growth compared to the wild type. In order to analyze the influence of both AOS isoforms on the sporophyte formation, Δ*PpAOS1* and Δ*PpAOS2* mutants were grown under sporophyte-inducing conditions [40]. For neither of the mutants an effect on sporophyte development and sporogenesis was observed since mutant spores were able to germinate like spores from wild type. In order to analyze molecular consequences of the AOS-disruption on the wound response of *P. patens* we analyzed the amount of *cis*(+)-OPDA formed by the moss upon wounding. For this purpose we monitored *cis*(+)-OPDA-accumulation in *P. patens* WT, Δ*PpAOS1* and Δ*PpAOS2* knock-out moss 1 h after a mechanical wounding stimulus employing LC/MS-analysis. Shown in Figure 8 are the results obtained for two independent experiments employing one line for each *PpAOS1* and *PpAOS2* knock-out. Although the over-all amount of *cis*(+)-OPDA differs in both experiments slightly, the obtained datasets demonstrate that the amount of *cis*(+)-OPDA is comparable in both WT and Δ*PpAOS2,* while that found in Δ*PpAOS1* was decreased by a factor of 5–12.

## Discussion

In the present study we aimed to analyze the biochemical and physiological properties of two AOS isoforms from *P. patens* and compared those with that of a previously characterized Cyp74 enzyme, PpHPL [18]. For this purpose all three recombinant enzymes were purified from *E. coli* and their biochemical parameters were analyzed. In addition, the sub-cellular localization of both AOS isoforms as well as the phenotype and the wound-induced *cis*(+)-OPDA formation of Δ*PpAOS1* and Δ*PpAOS2* knock-out lines was investigated.

In initial experiments we analyzed products formed from PpAOS2 and 9-HPOD via LC/MS. As our kinetic measurements demonstrated that the k_cat_-values of incubations of PpAOS2 with C_18_-derived substrates are much lower than that of other AOS including PpAOS1 (Table 2; discussed in detail below), we aimed to verify that PpAOS2 exhibits true AOS activity and thus results in formation of AOS products. As shown in Figure 5, these data demonstrate that like PpAOS1 also PpAOS2 exhibits AOS-activity and forms unstable allene oxide derivatives that decompose in aqueous solution yielding different ketol-isomers. PpAOS1 and PpAOS2 have a slightly acidic to neutral pH-optimum (PpAOS1, pH 6.5 vs. PpAOS2, pH 7.0). Recently, a similar pH-optimum was already determined for PpAOS1 [21]. Interestingly, the pH-optima for both PpAOS isoforms are consistent with that of the corresponding LOX isoforms [20], as the fatty acid hydroperoxide substrates for AOS activity are typically provided by LOXs [43]. The kinetic analyses of both AOS isoforms demonstrated that PpAOS1 can use both the 9- and 13-LOX-derived hydroperoxides from 18:2(n-6), 18:3(n-6) and 18:3(n-3) as well as 20:4(n-6)-derived 12-HPETE as substrate with a similar specificity (Table 1). Notably, the k_cat_-values determined for those incubations are comparable with those reported for AOS from other organisms [25,44]. Analogous analyses with PpAOS2 showed on the other hand that this enzyme exhibits only a very minor catalytic activity with all the used C_18_-derived substrates. With 12-HPETE as substrate, however, PpAOS2 showed a similar high activity as PpAOS1 pointing to a distinct specificity of PpAOS2 for C_20_–derived fatty acid hydroperoxides. This finding suggests that PpAOS2 may specifically be involved in the eicosanoid pathway in *P. patens*[19].

Recently, we found that similar to PpAOS1 also PpHPL can accept C_18_- as well as C_20_-derived hydroperoxides as substrates and convert them to short chain aldehydes and ω-oxo fatty acids. This enzyme, however, showed a preference for 9-hydroperoxides of C_18_-polyunsaturated fatty acids [18]. Interestingly, incubations of radio-labeled hydroperoxide isomers with both AOS isoforms not only led to the formation of ketols but also of short chain -oxo fatty acids (Figure 6 and Table 3). Ketols are typical AOS products that are formed by non-enzymatic hydrolysis of the highly instable allene oxide product [10,11]. Short chain -oxo fatty acids are, however, products that are specifically formed by the catalytic activity of HPL [45]. These results suggest that both PpAOS isoforms possess an inherent HPL activity. This finding is in line with previous studies on rice and *Arabidopsis* AOS [30] and reflects the close interconnection of the AOS and HPL catalytic pathway [25,29,30]. In a recent study a molecular determinant that connects these two activities has been identified. It is hypothesized that a particular Phe residue in the active site of AOS plays a central role in catalysis by stabilizing an intermediary formed carbon centered substrate radical. Mutational studies using AtAOS as a prototype enzyme showed that replacement of this residue by a HPL-specific Leu is sufficient to convert an AOS into a HPL [25,30]. However, until now this concept has only been evaluated by using AtAOS which has a preference for 13-hydroperoxide C_18_-fatty acids [37]. In order to analyze if this concept can also be applied on unspecific AOS, we generated a PpAOS1 variant with the respective Phe to Leu-substitution and analyzed the products formed by incubating purified protein with different 9- and 13-hydroperoxy C_18_-fatty acids as substrate. These data indeed showed that the PpAOS1_Phe93Leu variant has prominent HPL activity with all used substrates suggesting a similar substrate positioning with respect to the conserved Phe residue for both regio isomers in the wild type enzyme. We further evaluated whether the Phe/Leu-concept can also be applied on PpHPL. Sequence alignments showed that this particular AOS determinant is indeed conserved among the members of the Cyp74a-family (AOS) but interestingly also found in PpHPL (Phe-151). Based on the mechanism proposed for AOS-catalysis we hypothesized that this Phe residue in PpHPL might stabilize an intermediary formed carbon-centered substrate radical which would lead to allene oxide formation. When we probed incubations of PpHPL with different substrate isomers for AOS and HPL products, we indeed found beside ω-oxo fatty acid derivatives (HPL-products) also small amounts of other products (Figure 6 and Table 3). Among those we found both ketol isomers which might be formed by the inherent AOS activity of PpHPL. Interestingly, we observed the α-ketol being formed in lower amounts compared to the γ-ketol (Figure 6). Typically allene oxide hydrolysis leads to predominant formation of α-ketols while the amount of γ-ketols is significantly less [46]. However, Grechkin and co-workers showed that the trajectory of this unspecific reaction is highly pH-dependent and γ-ketol formation is favored at acidic pH [46] – reaction conditions that may apply to our experiments. The influence of this unusual Phe-151 in PpHPL on its product specificity was further analyzed. It is tempting to assume that substitution of AOS-specific Phe in HPL would increase HPL and decrease AOS activity. However, experiments using the respective PpHPL_Phe151Leu-variant showed an identical product pattern as for the wild-type enzyme. A similar result was obtained when a conserved HPL-specific Ala (Ala-169) was replaced by an AOS-specific Ser. This determinant has been reported to determine AOS/HPL activity as well. Neither the single variant (PpHPL_A169S) nor the double variant (PpHPL_F151L/A169S) showed an altered catalytic specificity (Table 3). These results suggest that in PpHPL residues other than those reported to be essential for AOS catalysis determine product specificity. As reported in supplemental material of [25], a similar result was obtained before. Here, an analogous enzyme variant of tomato HPL (LeHPL_L101F,A119S) exhibited only 5% AOS activity.

Recent studies showed that in contrast to flowering plants the moss *P. patens* may employ only the plastidic part of the oxylipin pathway, while the peroxisomal part is missing [16,23]. This observation is based on the findings that upon wounding or pathogen infection only *cis*(+)-OPDA accumulated in the moss, but no JA could be detected. It was further supported by immunocytological investigations indicating the plastidic localization of all LOX and AOC forms [16]. We here provide supporting evidence by showing that also PpAOS2 is localized in the plastid (Figure 7). Surprisingly, however, PpAOS1 appeared to be merely localized in the cytosol. As mentioned above AOS products are highly unstable in aqueous solution and decompose within a few seconds either by non-enzymatic hydrolysis or spontaneous cyclization to ketols or cyclopentenones [11]. Based on this finding and on the fact that natural occurring OPDA is enantiopure in the *cis*(+)-configuration [47] several studies discussed (for review *cf.*[8]) a physical coupling of AOS and AOC *in vivo*. Although a later study demonstrated that no physical interaction of both enzymes *in vitro* is necessary in order to yield *cis(+)*-OPDA [48], it appeared highly questionable to us if *cis(+)*-OPDA can be formed by the action of spatially separated PpAOC1/2 and PpAOS1. On the other hand our kinetic analysis showed that PpAOS2 has a distinct specificity for C_20_-derived substrates and thus may not be capable of providing the PpAOCs with the substrate needed for *cis*(+)-OPDA-formation. In order to tackle this issue we generated Δ*PpAOS1* and Δ*PpAOS2* knock-out mutants, analyzed the *cis*(+)-OPDA accumulation upon wounding and compared these results with those obtained for the wild type strain. These analyses showed that formation of *cis*(+)-OPDA was highly impaired by the disruption of PpAOS1, but not by the disruption of PpAOS2. While this fact therefore indeed indicates that in contrast to PpAOS2, PpAOS1 plays a major role in the biosynthetic pathway of *cis*(+)-OPDA and thus is in line with our kinetic data, the spatial separation of PpAOS1 and PpAOCs raises the question of how the unstable PpAOS1 product is translocated from the cytosol to the plastid without being hydrolyzed. As mentioned previously [49] the allene oxide might be protected from hydrolysis by an pure hydrophobic environment; thus one may speculate that AOS products are imbedded into the membrane matrix *in planta* and are subsequently translocated by a so far unknown mechanism to the plastid where they may serve as substrate for PpAOCs. A further question that is raised by the fact that both AOS are differentially localized concerns the physiological function of those PpAOSs. In flowering plants it has been shown that analogous knock-out mutants suffer from defective anther and pollen development and are thus male sterile [50] or have defects in egg cell development and are thus female sterile [51]. In addition, previous studies on Δ*PpAOC* knock-out mutants showed in analogy to studies in flowering plants [50,51], that reduction of *cis*(+)-OPDA biosynthesis leads to reduced fertility, aberrant sporophyte morphology or interrupted sporogenesis [16]. In contrast, neither the Δ*PpAOS1* nor the Δ*PpAOS2* knock-out mutants described here showed any obviously deviating phenotype in growth or sporophyte development. While this finding is consistent with the cytosolic localization of PpAOS1, it is surprising that deletion of PpAOS2 also did not show any phenotypic effect. Thus, the finding that each AOS knock-out mutants show no aberrant phenotype suggests that *cis*(+)-OPDA is still being synthesized in sufficient amounts. On the one hand this may be explained by overlapping functions of PpAOS1 and PpAOS2. However as only PpAOS2 is localized in the plastid and thus can provide PpAOCs with allene oxide substrate, a redundant function of both enzymes appears to be unlikely. Also, a careful re-investigation of the genome did not reveal a further potential AOS-sequence. On the other hand, an inherent AOS activity was observed for PpHPL. Considering the high substrate turnover of PpHPL [18], its AOS side activity may still be efficient enough to provide sufficient amount of allene oxide product for formation of *cis*(+)-OPDA by PpAOC. In line with this assumption goes the plastidic localization of PpHPL [18].

## Conclusions

We found that both AOS isoforms from *P. patens* are capable of metabolizing C_18_ and C_20_-derived fatty acid hydroperoxides with different specificities suggesting that both enzymes might have different substrate pools. In line with that, only PpAOS2 is localized in the plastid where oxylipin metabolism takes place and PpAOS1 was detected in the cytosol. Surprisingly, however, only disruption of PpAOS1 affected wound response and led to an decreased formation of *cis*(+)-OPDA. Furthermore, knock-out mutants of neither AOS showed an aberrant phenotype suggesting that there are overlapping functions with the other Cyp74 enzyme, PpHPL. This is supported by site directed mutagenesis experiments. These revealed that the catalytic trajectories of substrate unspecific PpAOS1 and PpHPL are closely inter-connected and can be inter-converted by single amino acid exchanges.

## Methods

All chemicals used in this study were either from Sigma-Aldrich or from Carl Roth & Co. Agarose was purchased from Biozym Scientific GmbH while all fatty acids were from Cayman Chemicals. Acetonitrile was from Fisher Scientific and restriction enzymes were purchased from MBI Fermentas.

### Cloning, expression and site directed mutagenesis of PpAOS1, PpAOS2 and PpHPL

Both AOS genes were identified based on the sequence similarity towards plant Cyp74-enzymes in an EST-library described in [18]. Full-length cDNA was obtained by 5′-RACE using a lambda ZAPII cDNA-library of *P. patens* protonema. PpHPL was PCR-amplified from the construct reported before [18]. Resulting PCR-fragments were cloned into the pGEM-T vector (Promega) using primers with *Sph*I/*Xho*I (PpAOS1) and *Nhe*I/*Hind*III (PpAOS2) restriction sites. In order to increase enzyme solubility we added recombinantly the peptide sequence MAKKTSS to the N-;terminus of PpHPL as described before [25]. For heterologous expression *PpAOS1* was cloned into the pQE30-vector (Qiagen) and transfected into *E. coli* SG13009 [pRep4] while *PpAOS2* and *PpHPL* were cloned into the pET28a and pET24b vectors (Invitrogen), respectively, and transfected into *E. coli* Bl21star. Recombinant cells bearing the respective plasmid were incubated in LB or 2xYT medium until cells reached an OD_600_ 0.6 - 0.8. In order to assure that heme production was not the limiting factor during enzyme expression we also added 80 mg/L α-amino levulinoic acid and 150 μM 0.1 mM ammonium iron citrate. Protein expression was induced by the addition of 1 mM isopropyl β-D-thiogalactopyranoside and cells were incubated for 3 d at 16°C. PpHPL was expressed as described previously [18]. Enzyme variants with defined single point mutations were generated from the respective plasmids by employing the site directed mutagenesis technique using the Phusion™-“High-Fidelity”-PCR-System (Finnzyme) according to the manufactures instructions.

### Transient expression of YFP C-terminal fusion constructs

EYFP from plasmids carrying the authentic clones were provided by Martin Fulda (Georg-August-University, Göttingen, Germany). EYFP cDNA was cloned into the plasmid pUC18-Entry [52] via PCR using primers with *Not*I and *Sal*I restriction sites resulting in pUC18-Entry-YFP. *PpAOS1* and *PpAOS2* cDNA were introduced into pUC18-Entry-YPF using primers with *EcoR*V and *Not*I restriction sites. Via Gateway LR Clonase Mix (Invitrogen) the *PpAOS1-YFP* and *PpAOS2-YFP* coding sequence was transferred into a modified pCAMBIA33.1 plasmid, a plant expression destination vector containing the cauliflower mosaic virus (CaMV) *35S*-promoter, an *att*R1/R2 gateway cassette and a CaMV-*35S*-terminator [52]. Onion epidermal cells and *P. patens* gametophores on agar plates were transformed via particle bombardment using plasmid-coated 1 μm gold particles with a helium-driven particle accelerator (PDS-1000/He; BioRad), 350 psi rupture disks and a vacuum of 27 inches of mercury. Gold particles were coated with 4 – 8 μg of plasmid-DNA. After bombardment the onion cells were incubated for 14 – 20 h at RT and gametophores were cultivated under long light conditions (16 h of light, 8 h of darkness). In addition, constructs were expressed in moss protoplasts as described before [18]. Images were recorded using an Olympus BX51 epifluorescence microscope or a Zeiss LSM 510 confocal microscope.

### Generation of *P. patens* targeted gene knockout mutants

The transfection of gene-disruption constructs into *P. patens* protoplasts and the regeneration of transgenic lines was performed according to standard procedures [39]. For generating the Δ*PpAOS1* knock-out mutant, the selection marker cassette (*nos*-promoter::neomycin phosphotransferase::*nos*-terminator) derived from the vector pBIN19 [53] was cloned into the *Xcm*I site of the cDNA-pGEM-T-subclone. The Δ*PpAOS2* knock-out mutant was generated by first cloning the selection marker cassette (CaMV-*35S*-promoter::neomycin phosphotransferase::CaMV-*35S*-terminator) derived from pCAMBIA2300 (accession nr. AF234315.1) into pUC18, then subcloning a 5^′^*PpAOS2* fragment of 620 bp using *EcoR*I/*Bgl*II restriction sites and finally subcloning a 3^′^*PpAOS2* fragment of 590 bp using *Sal*I/*Hind*III restriction sites. Before transfection the gene disruption constructs were released from the vector backbone by digestion with suitable restriction enzymes (*PpAOS1*: *Hind*III/*Apa*I; *PpAOS2*: *Hind*III/*Ase*I). Targeted gene knockout lines were identified by PCR using DNA extracts prepared from transgenic *P. patens* lines using a modified CTAB-protocol [54]. Clones of two independent PpAOS1 and five independent PpAOS2 knockout mutants have been stored over liquid nitrogen and are made freely available via the International Moss Stock Center IMSC (http://www.moss-stock-center.org/) with the following accession numbers: Δ*PpAOS1* knock-out mutants: IMSC 40383 (KO5), IMSC 40384 (KO21); Δ*PpAOS2* knock-out mutants: IMSC 40686 (KO9), IMSC 40687 (KO14), IMSC 40688 (KO71), IMSC 40689 (KO77) and IMSC 40670 (KO119) [55].

### Growth conditions

Gametophores were grown under standard conditions, resulting in the formation of colonies within 4 weeks. After that the moss was grown under sporophyte inducing conditions (culture conditions are detailed in [40]).

### Analysis of cyclopentenones

The analysis of cyclopentenones was performed as described in [42] for phytohormone determination with one modification. For detection of OPTA the following MRM transition was added: 317/273 [declustering potential (DP) −65 V, entrance potential (EP) −4 V, collision energy (CE) −22 V].

### Cell lysis and protein purification

Cells were lysed in accordance to the procedure of [33], with some modifications: Briefly, cells from 1 L culture were harvested by centrifugation (8 000 × g, 20 min, 4°C) and the resulting cell paste was dissolved in 150 mL 100 mM Tris/HCl (pH 7.8) containing 20% glycerol. Lysozym was added (0.2 mg/mL) and incubated for 30 min at 4°C. After centrifugation (8000 × g, 10 min, 4°C) the sedimented spheroblasts were dissolved in 50 mL 100 mM sodium phosphate (pH 8.0) buffer containing 14 mM magnesium acetate, 60 mM potassium acetate, 0,1 mM DTT, 500 mM urea and frozen for 18 h at −80°C. The protease inhibitor phenylmethylsulfonylfluoride was added to a final concentration of 0.5 mM and cells were lysed by employing a sonifier cell disrupter (B15) from Branson. Cell debris were removed by centrifugation for 20 min at 50 000 × g and 4°C. The resulting cell free extract was applied on immobilized Ni^2+^-column (His-Trap™ HP-column from GE-Healthcare) by employing an ÄKTAprime system. Unspecific bound proteins were eluted with 50 mM sodium phosphate buffer (pH 8.0) containing 50 mM NaCl, 500 mM urea. In case of PpAOS2-purification we applied an additional step, in which we washed the column with 50 mM sodium phosphate buffer (pH 8.0) containing 50 mM NaCl, 500 mM urea and 15 mM imidazol in order to further elute unspecifically bound proteins. Finally, elution of specifically bound proteins was performed in 50 mM sodium phosphate buffer (pH 8.0) 1 M NaCl, 500 mM urea and a linear gradient from 50 mM - 300 mM imidazole within 20 min and a flow rate of 1 mL/min. The purity of the eluted protein was assessed by SDS-PAGE analysis [56].

### Kinetic properties and pH-optimum

Initial experiments aimed to determine the optimal pH for the enzymatic conversion of 30 μM 13*S*-HPOD catalyzed by both AOS-isoforms. For this purpose we measured the time dependent decrease of absorption at 234 nm at a given pH employing a Cary 100 Bio spectrophotometer (Varian). The reaction was carried out at room temperature in different buffer systems with defined pH (200 mM sodium acetate (pH 4.7 - 5.5), 200 mM sodium phosphate (pH 5.5 - 8.0), 200 mM sodium borate (pH 8.0 - 10.5)) and started by the addition of 100 nM AOS. Kinetic properties were determined in analogous experiments by analyzing the time dependent decrease in absorption at 234 nm for different substrate concentrations. Typically we used concentration ranging from 2 μM - 100 μM. Substrates for which we found high K_M_-values, were used with concentrations of up to 150 μM. The different substrates used for this analysis were: 9- and 13-hydroperoxy derivatives of 18:2(n-6) (9-/13-HPOD), 18:3(n-6) (9/13-HPOT(n-6), 18:3(n-3) (9/13-HPOT(n-3)) as well as the 12-hydroperoxy derivative of 20:4(n-6) (12-HPETE). Note that different enzyme concentrations were applied in these experiments: PpAOS1-concentrations were 0.05 nM for incubations with 9/13-HPOT(n-6) and 0.1 nM for incubations with 9/13-HPOD, 9/13-HPOT(n-3) and 12-HPETE. PpAOS2-concentrations for incubations with 12-HPETE were 1 nM and 100 nM for experiments with 9/13-HPOT(n-6), 9/13-HPOD and 9/13-HPOT(n-3). For the calculation of K_cat_ values we took into account, that the heme-occupancies of PpAOS1 and PpAOS2 were 30% and 4%, respectively.

While the 9 and 13-isomers were prepared from incubation of the respective fatty acid with potato LOX or soybean LOX, respectively, 12-HPETE was formed from 20:4(n-6) by using *P. patens* LOX1 and 2 [20]. These procedures have been described elsewhere [18].

### Product analysis

All substrates used for product analysis were ^14^C-labeled at the C-1 and prepared similar to the method described above. Enzymatic conversions of the [1-^14^C]-hydroperoxy fatty acid substrates were typically performed in 1 mL 100 mM sodium phosphate buffer (pH 6.0) at room temperature under constant shaking for 30 min. Formed products were extracted with diethyl ether and analyzed with by RP-HPLC employing a 1100HPLC system (Agilent) that was equipped with a LiChroCART® 125–4 LiChrospher®100-RP-18 (5 μm) column (Merck) similar to method described before [57]. Briefly: a solvent system that consisted of acetonitril/water/acetic acid (50/50/0.1, v/v/v) as solvent system A and acetonitril/water/acetic acid (80/20/0.1, v/v/v) as solvent system B was used. The gradient elution profile employed in this study was: flow rate: 1 mL/min, 0–25 min, 100% A; 25–26 min from 100% A to 100% B; 26–36 min, 100% B, 36–38 min from 100% B - 100% A. Radio-labeled products were detected with a Raytest radio detector that was coupled to the chromatographic device. All products formed were confirmed by LC-MS analysis as described before [58].

## Abbreviations

AOC: Allene oxide cyclase; AOS: Allene oxide synthase; HPL: Hhydroperoxide lyase; DES: Divinylether synthase; P450: Cytochrome P450; YFP: Yellow fluorescent protein; LOX: Lipoxygenase; EOD: Epoxy octadecadienoic acid; EOT: Epoxy octadecatrienoic acid; H(P)ETE: Hydro(pero)xy eicosatetraenoic acid; HPOT: Hydroperoxy octadecatrienoic acid; HPOD: Hydroperoxy octadecadienoic acid; JA: Jasmonic acid; OPDA: *cis*(+)-12-oxo phytodienoic acid; OPTA: 11-oxo prostatrienoic acid; RP: Reversed phase; PCR: Polymerase chain reaction; WT: Wild type; HPLC: High pressure liquid chromatography.

## Competing interests

The authors declare that they have no competing interests.

## Authors’ contributions

JS, EH, BF, WF and MS cloned and mutated different Cyp74 constructs. JS isolated and purified proteins and performed incubation experiments. JS, CH, AKB, BF and FB performed the product analysis. Localization experiments were carried out by EH. FB, EH, WF, RR and IF designed the research and wrote the paper. All authors read and approved the final manuscript.

## Supplementary Material

Additional file 1 **Figure S1.** Kinetic analysis of PpAOS1 (A) and PpAOS(2) with 9-HPOT(n-3) as substrate. The reaction was started by the addition of enzyme and the time dependent changes at 234 nm were monitored spectrometrically. Data were fitted to the Michaelis-Menten equation. Click here for file
