# Supplementary material for: Biosynthesis of allene oxides in Physcomitrella patens
Source: BMC Plant Biol. 2012 Nov 30;12:228. doi: 10.1186/1471-2229-12-228 (PMC3552686; doi:10.1186/1471-2229-12-228)

Supplemental Material:

Supplemental Figure S1: Kinetic analysis of PpAOS1 (A) and PpAOS(2) with 9-HPOT(n-3) as substrate. The reaction was started by the addition of enzyme and the time dependent changes at 234 nm were monitored spectrometrically. Data were fitted to the Michaelis-Menten equation.

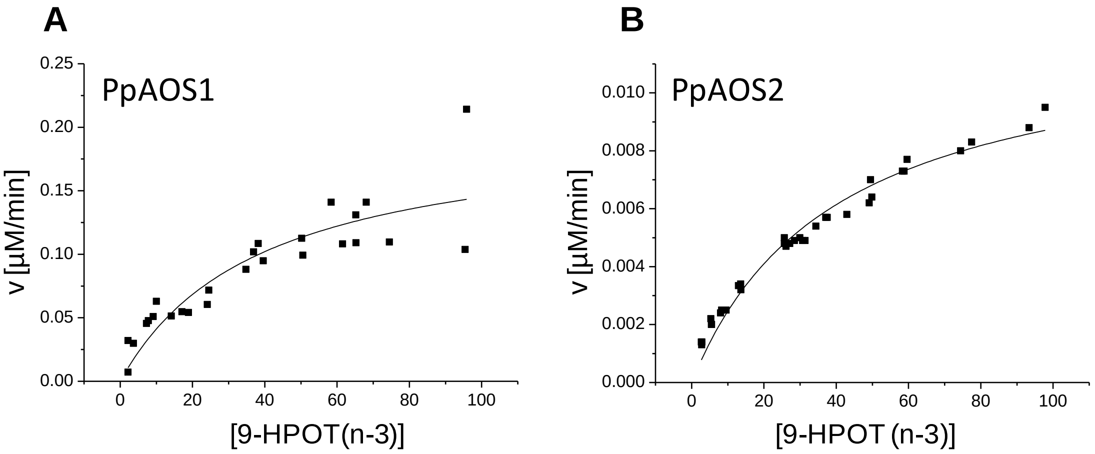

Supplement: Additional file 1 — Figure S1. Kinetic analysis of PpAOS1 (A) and PpAOS(2) with 9-HPOT(n-3) as substrate. The reaction was started by the addition of enzyme and the time dependent changes at 234 nm were monitored spectrometrically. Data were fitted to the Michaelis-Menten equation. [file 1471-2229-12-228-S1.pdf]
